# Immigrant Naturalisation, Employment and Occupational Status in Western Europe

**DOI:** 10.3389/fsoc.2020.00070

**Published:** 2020-12-16

**Authors:** Rezart Hoxhaj, Maarten Vink, Tijana Breuer

**Affiliations:** ^1^Migration Policy Centre, Robert Schuman Centre for Advanced Studies, EUI, Florence, Italy; ^2^Department of Political Science, Maastricht University, Maastricht, Netherlands; ^3^Maastricht University School of Business and Economics, Maastricht, Netherlands

**Keywords:** citizenship, employment, occupational status, Western Europe, citizenship policy

## Abstract

Does citizenship facilitate access to employment and higher status jobs? Existing studies have produced mixed results across mostly single case studies in Europe and North America. To investigate whether this heterogeneity depends on varying institutional and socio-economic conditions, in this paper we analyse the labour market outcomes of immigrants who have naturalised in 13 West European countries. Our empirical analysis draws on data from the 2014 European Labour Force Survey *Ad Hoc* Module on immigrants. In order to cope with the selective nature of the naturalisation process, we employ a bivariate probit model that accounts for unobserved characteristics of naturalising immigrants. Our main results show a positive relationship across these destination countries between citizenship and the probability of employment, as well as between citizenship and occupational status, but only for immigrant men from developing countries. For women and for migrants from developed countries, we observe no significant differences between citizens and non-citizens. Liberalising the access to citizenship does not diminish the positive returns on employment from naturalisation. For immigrant men from developing countries there is evidence of a trade-off between easier access to citizenship and the returns on occupational status.

## Introduction

Citizenship acquisition is often viewed as a vehicle for the labour market integration of migrants. Acquisition of citizenship is mainly associated with better employment chances, higher earnings and higher occupational positions (Liebig and Von Haaren, 2011; Hainmueller et al., 2019). Over the past 15 years, various studies have been published drawing on data from surveys, census and population registers in Europe and North America (e.g., Bratsberg et al., 2002; DeVoretz and Pivnenko, 2005; Scott, 2008; Fougère and Safi, 2009; Rallu, 2011; Bevelander and Pendakur, 2012; Steinhardt, 2012; Helgertz et al., 2014).

Yet it is hard to draw general conclusions from these studies, given that there is considerable variation in terms of national context, the dependent variable and the type of data available (for overviews, see Steinhardt, 2012, p. 815, 816; Helgertz et al., 2014, p. 343). While the variability of the effect of citizenship acquisition on labour market outcomes has often been noted (e.g., Liebig and Von Haaren, 2011, p. 17, 18), there has been surprisingly little systematic attention to the question to what extent this heterogeneity is due to differences in contexts of study. This lack of attention for the relevance of contextual factors is particularly striking, given that the citizenship policy of the destination country is a strong predictor of the likelihood of immigrants, especially from less developed parts of the world, to acquire the citizenship of a developed destination country (Vink et al., 2013).

In this paper we propose a comparative approach to the analysis of the so-called “citizenship premium” in the labour market. We aim to answer the following two questions: first, to what extent do the often-observed positive associations between citizenship and, respectively, employment and occupational status hold across a larger set of destination countries in Western Europe?; and, second, to what extent does the citizenship policy of the destination country, condition these relationships by facilitating or restricting the access to citizenship?

Given the selective nature of the naturalisation process, where an effect of citizenship can be identified, it may well be caused by characteristics inherent in the group of migrants that naturalises rather than in the status of citizenship itself (for an early discussion, see Chiswick, 1978). In order to cope with the selective nature of the naturalisation process, we employ in this paper a recursive bivariate probit model and the treatment effect method that account for unobserved characteristics of naturalising immigrants. By doing so, we provide more robust comparative evidence on the association between citizenship, on the one hand, and employment and occupational status, on the other, compared to previous studies that do not take into account this selectivity (in particular, Zwysen, 2018).

We analyse this question by means of the 2014 *Ad Hoc* Module of the European Labour Force Survey on the labour market situation of migrants and their immediate descendants, which offers cross-national comparative information on citizenship status, labour market status, and a range of other characteristics of foreign-born residents in Europe. We focus exclusively on foreign-born residents in 13 West European countries and look at the probability of having paid employment and having a higher-status job.

This paper is organised as follows. In section State of the Art we outline the theoretical framework of our paper, by first (section Migrant Disadvantage in the Labour Market and the Citizenship Premium) discussing existing theories on the effect of citizenship on labour market outcomes (“the citizenship premium”) and, subsequently, discussing theoretical arguments for why the effect of citizenship may be conditioned by citizenship policies, i.e., by the relative facilitated or restricted access to citizenship. Section Data and Methodology describes the data and methodology employed in the analysis. The key findings are presented in section Results and some conclusive remarks are presented in section Conclusion.

## State of the Art

### Migrant Disadvantage in the Labour Market and the Citizenship Premium

There is substantial evidence that employment levels, occupational status and wages significantly differ between first—and even second—generation migrants and natives in all of the western economies (Borjas, 1994; Kogan, 2006; Heath and Cheung, 2007; Fleischmann and Dronkers, 2010; Yann et al., 2010; Lancee, 2012). The current literature identifies a number of reasons why first-generation migrants face disadvantages in the labour markets of the developed countries to which they have immigrated. In the first place, migrants are often endowed with lower levels and different kinds of human capital than those that are necessary to fare successfully in western labour markets (Heath and Cheung, 2007). This is especially the case of migrants from less developed countries who have grown up in challenging socio-economic circumstances with limited educational opportunities. In the second place, the majority of first-generation migrants in Western Europe lack mastery of the language of the country of destination (Van Tubergen and Kalmijn, 2005; Heath and Cheung, 2007). This lack of knowledge reduces their potential productivity and consequent employability in many segments of the labour market. Thirdly, migrants' educational credentials obtained in their country of origin may not have the same value in the labour markets in their countries of destination, as employers are often unable to evaluate foreign qualifications and therefore prefer domestic qualifications with known interpretations in terms of skills and productivity. Additionally, various restrictive practices and regulations exclude first generation migrants from performing certain types of jobs; a notable example of such a restriction is the requirement of citizenship for public sector job entry. Finally, labour market experience obtained in the country of origin is not easily transferable, nor equally valuable in the labour market in the country of destination (Heath and Cheung, 2007; Chiswick and Miller, 2009). While lack of human capital embodied in skills and labour market experience is seen as the major cause of the labour market disadvantage among the first generation of ethnic minorities in Europe, migrants are also affected by prejudice and discrimination (André et al., 2009). A lack of knowledge of, or familiarity with, migrants' socio-economic background makes employers reluctant to hire them for both rational and irrational reasons. While it is indeed difficult to objectively judge migrants' potential productivity (rational discrimination), some employers often prefer one ethnic group over another even if the expected productivity of the two groups is the same (irrational discrimination) (Fougère and Safi, 2009).

In this context of migrant disadvantage in labour markets, access to citizenship is seen as one of the focal points of public policy aimed at promoting migrant integration. Generally, literature has reached a consensus on the positive effect of citizenship on employment (Fougère and Safi, 2009; Corluy et al., 2011; Bevelander and Pendakur, 2012; Engdahl, 2014; Gathmann and Keller, 2018), though some studies observe no effect (Bevelander and DeVoretz, 2008) or even a negative effect (Scott, 2008). Three main mechanisms behind the assumed link between citizenship and successful labour market integration are identified (Liebig and Von Haaren, 2011; Hainmueller et al., 2019; Peters et al., 2020). First, citizenship eliminates barriers to public sector jobs and to a range of regulated high-skill professions or self-employment (Gathmann and Keller, 2018 in the case of Germany). Moreover, naturalisation eliminates barriers to some other jobs that require unrestricted mobility of their employees without any bureaucratic hurdles. This aspect is particularly relevant for non-EU immigrants who need a visa to travel inside and outside of Europe (Steinhardt, 2012; Poeschel, 2016)^1^. More generally, it will be more attractive for employers to hire naturalised migrants as the administrative costs of hiring and retaining foreign-born workers will be lower in the case of those who hold destination citizenship.

Second, it has been argued that the acquisition of citizenship increases the employability of first-generation migrants by signalling successful integration to employers (the signalling argument). As outlined above, it is often difficult for employers to judge the potential productivity of foreign workers due to their unfamiliarity with the “standard” indicators of productivity, such as educational qualifications and work experience, but also their general commitment to a job. For this reason it has been argued that citizenship might serve as a device signalling “good” integration, leading employers to assume that those migrants who acquire citizenship have higher levels of productive skills, and also a commitment to invest in the country-specific human and social capital. Consequently, the signal of long-term commitment may induce employers to lower barriers to training (von Haaren-Giebel and Sandner, 2016) or to career mobility of immigrants within the firm. Previous research suggests that the citizenship premium is stronger for migrants who face the highest structural barriers in the labour market, especially those from economically less developed parts of the world (Bratsberg et al., 2002, p. 590; Fougère and Safi, 2009, p. 138; Peters et al., 2020).

Third, naturalisation may encourage long-term commitment to the destination country labour market and hence induce migrants' human capital development (Bratsberg et al., 2002, p. 572), for example by investment in mastery of the native language or obtaining country-specific diplomas (or going through often arduous processes of diploma recognition) that provide access to regulated professions. This human capital perspective relates to sociological literature in which a realistic perspective on naturalisation leads migrants to view naturalisation as a logical step in their trajectory of building up a life in the host country (Aptekar, 2015, p. 65). Crucially, such a view implies that labour market effects may be observed not just *after* the moment of acquiring citizenship (as would be the case in the “signalling” argument), but also *before* naturalisation, as employment propensity and wages are likely to increase in conjunction with human capital acquisition (Bratsberg et al., 2002; Peters et al., 2018, 2020).

While the citizenship premium in terms of access to employment is relatively well investigated by the literature, few studies exist on the relationship between citizenship and upward occupational mobility. Bratsberg et al. (2002) show that white-collar and public-sector employment rates are higher for those who naturalise in the U.S than for those who do not. They argue that this effect was not due to the increased human capital investment before naturalisation but mainly because naturalisation increases access to preferred jobs. According to Jarreau (2015), naturalisation enhances job mobility, both the change of occupations and employers, and reduces job mismatching. Euwals et al. (2010) on Turkish immigrants in Germany and Netherlands find a positive effect of citizenship on occupation status, whereas Kogan (2003) finds a negative effect of citizenship on ex-Yugoslav immigrants in Austria and a not significant effect in Sweden. Finally, using the EU-LFS (2008) *ad hoc* module, Zwysen (2018) studies whether the acquisition of citizenship—intended as a proxy for host country human capital—affects the labour market integration of immigrants. This study finds a slightly positive association of naturalisation with job quality but not with employment. However, this study does not take into account the selection of immigrants into citizenship.

### The Citizenship Premium Across National Contexts

Given the heterogeneity in findings observed in the literature with respect to the citizenship premium in the labour market, not just with regard to migrant groups but also with regard to the context of study in various publications, the question arises to what extent migrants experience higher employment probability and have access to higher status jobs after naturalisation across various national contexts. We argue in this paper that at least one important contextual aspect—citizenship policies—could be expected to condition the relationship between naturalisation, on the one hand, and employment and occupational status, on the other.

Citizenship policies in Europe differ substantially, reflecting not only the fact that this is one of the last bastions of sovereignty, but also historically rooted approaches to membership and belonging (Vink and de Groot, 2010). Naturalisation requirements in particular vary greatly, with for example 5 years of residence required in countries such as France, the Netherlands, Sweden and the United Kingdom and 8–10 years in others, such as Austria, Germany and Italy. As a consequence, we see large differences in citizenship take-up rates, with around 80 percent of the foreign-born population naturalised after at least 10 years of residence in the Netherlands and Sweden, but only around 35 percent of a comparable group in Germany and Switzerland (Liebig and Von Haaren, 2011).

There are contrasting theoretical arguments on how easier/faster access to citizenship might influence the citizenship premium. One perspective builds on the assumption that the extent to which citizenship functions as a signal of integration and commitment to the host society is largely determined by the way society in general, and employers in particular, perceive the value and meaning of citizenship. From this perspective, liberal citizenship policies might “devalue” citizenship in the eyes of employers and, thus, be less useful as a selection device between migrants, because the acquisition of citizenship is relatively easy in terms of naturalisation conditions and procedure (see, notably, Koopmans, 2010). In other words, if it is perceived to be “normal” to have citizenship (i.e., the majority of the foreign-born population has citizenship of the country of destination), then having citizenship might not be perceived as a signal of integration, but merely a direct consequence of liberal policies. Peters et al. (2020, p. 532) in a study on the labour market effects of naturalisation in the Netherlands observe in this context, that the signalling effect of the host country citizenship is stronger when access to the status is more exclusive. In this case, we do not expect employers to regard migrants with citizenship as being better integrated than those without. In line with our previous argument that citizenship is of most importance to those migrants who face the highest structural barriers in the labour market, this should particularly affect those immigrants from less developed parts of the world.

An alternative perspective on the relationship between citizenship policy and the citizenship premium argues that if citizenship is easily accessible in a country and consequently observed as such by employers, then the implicit expectation is that long-term resident immigrants should have citizenship. In this case, employers could assume that immigrants who have resided in a country for a number of years, but have not naturalised, hold unobservable negative characteristics. For example, employers could assume that those who have not naturalised do not have the necessary language skills to pass a citizenship test or that they are not committed to staying and integrating in the country of destination. Hence, in countries with liberal policies this would be “negative signalling.” If this is the case, then migrants without citizenship will be negatively selected in countries with liberal citizenship policies.

In contrast, easier/faster access to citizenship might incentivise immigrants to invest in education and in country-specific human capital in order to reap the benefits of naturalisation for a longer period (Gathmann and Keller, 2018). This is mostly true when citizenship gives access to a category of jobs that require specific skills and training and in contexts where severe labour market segregation of immigrants exist. Moreover, Hainmueller et al. (2016) also point to a psychological component according to which a faster naturalisation process makes immigrants feel more welcome and have them identify with the culture of the destination country. This could be a catalyst for a faster integration in the labour market and society. According to these arguments, in countries with liberal citizenship policies the positive effect of citizenship on the labour market outcomes of immigrants will be higher.

In sum, given the contrasting findings in the literature, the way citizenship policy may condition the citizenship premium becomes an empirical question that we will try to answer in this paper.

## Data and Methodology

### Data

For our empirical analysis, we use a special version of the European Labour Force Survey (EU-LFS), namely the EU-LFS *ad hoc* module (AHM) for 2014 on the labour market situation of migrants and their immediate descendants. The EU-LFS provides standardised cross-sectional data on labour market status and core demographic and migration information. The AHM 2014 provides additional information on the possible explanatory factors of migrant integration in Europe, such as country of birth of both parents, reason for migration, timing of naturalisation and an evaluation of migrants' qualifications. From the 27 countries covered by the EU-LFS AHM 2014, we included in the analysis 13 Western European countries having information on crucial variables used in the analysis^2^.

Our analysis focuses on foreign-born individuals between 22 and 64 years old residing in private households. We focus on “first generation” migrants because in this paper we aim to theorize and measure the link between the explicit decision to naturalise and the labour market outcomes of immigrants. As shown elsewhere, the questions of the acquisition of citizenship by the immediate descendants of migrants and that of their socio-economic integration are essentially different (Dronkers and Vink, 2012; Vink et al., 2013). In order to exclude as much as possible migrants who may have acquired destination country citizenship by descent, we only include individuals who themselves *and* both of whose parents were born outside the survey country. In addition, to exclude cases where migrants arrive at a young age and acquire destination country citizenship by extension of the act of naturalisation of their parents (rather than as an individual decision), we only include individuals who were at least 22 years old on arrival. Finally, we consider in our baseline analysis only those individuals who are eligible to naturalise, based on the years they have spent at destination at the time of the survey and the residence requirement for ordinary naturalisation in a country. We are not able to identify those immigrants who are married with citizens and may have facilitated access to citizenship through a shorter residency requirement. This means that for those immigrants who are married to a native citizen the effect of naturalisation on labour market outcomes may be confounded by the effect of interethnic marriage. Due to data limitations we cannot disentangle these effects in this study (see e.g., Peters et al., 2020 for an approach based on register data that allows greater precision in identifying eligibility, though only in a single country study). Supplementary Tables 4, 5 present some descriptive statistics of the sample we use for the empirical analysis by gender and the distribution of immigrants by country of destination, respectively.

### Estimation Strategy

The literature points out that the effect of naturalisation on labour market outcomes could be biased because unobserved individual characteristics, such as inherent ability or commitment, may affect both naturalisation choice and the labour market outcomes^3^. Consequently, it is difficult to disentangle the effect of naturalisation from pre-existing differences in these characteristics. To attenuate this typology of bias we estimate simultaneously a system of 2 equations; each outcome equation (the probability of having employment and the occupational status) with the probability of being naturalised equation (Equation 1 below, selection equation henceforth) (Fougère and Safi, 2009). We use the recursive bivariate probit method for the employment equation (Equation 2 below) and the treatment effect method (Maddala, 1983) for the occupational status equation (Equation 3 below)^4^. These methods allow the binary dependent choice (citizenship) in Equation (1) to be an endogenous regressor in Equations (2, 3). In our specification, we assume that identification of the parameters is possible without using an exclusion restriction and can be achieved by the functional form. Wilde (2000) argues that identification by the functional form is possible provided there is sufficient variability on the exogenous regressors. Other literature points out that the use of an exclusion restriction is a first best solution to address a possible failure of identification (Jones, 2007; Mourifié and Méango, 2014). In our case, an exclusion restriction is absent as employment outcomes and naturalisation are determined by the same variables. This is one of the methodological limits of our study.

(1) $$\begin{array}{l} Citizenship_{i}=\beta_{0}\;{+\;\beta }_{1}Z_{i}+\beta_{2}AreaOrigin_{j}\\ \;+\beta_{3}MigReason_{i}+\phi_{c}+\varepsilon_{i} \end{array}$$

(2) $$\begin{array}{l} Employed_{i}=\theta_{0}+\theta_{1}Citizenship_{i}+\theta_{2}Z_{i}\\ \;+\theta_{3}AreaOrigin_{j}+\theta_{4}MigReason_{i}\\ \;+\phi_{c}+\varepsilon_{i} \end{array}$$

(3) $$\begin{array}{l} OccupationalStatus_{i}=\delta_{0}+\delta_{1}Citizenship_{i}+\delta_{2}Z_{i}\\ \;+\delta_{3}AreaOrigin_{j}+\delta_{4}MigReason_{i}\\ \;+\phi_{c}+\varepsilon_{i} \end{array}$$

The dependent variable in selection Equation (1) is citizenship status, equal to 1 if the individual is a citizen of the country of destination and 0 otherwise. In the outcome Equation (2) the dependent variable is dichotomous indicating whether the respondent is currently employed or not^5^. The dependent variable in the outcome Equation (3) is a continuous variable (ISEI scale by Ganzeboom and Treiman, 1996) measuring the occupational status of individuals^6^. A higher occupational status score is associated to a higher prestige of the job. Note that our explanatory variable is *Citizenship*_*i*_ which enters as a dummy variable in the outcome equations.

The vector *z*_*i*_ includes the following individual-level variables: *Age* and *Age squared* measured in years; *Years of residence* and *Years of residence squared* measured as number of years in the destination country; 3 dummies for marital status (*Single, Married, Divorced/Separated*); 3 dummies measuring the level of educational attainment (*High education, Medium education, Low education)*; 4 dummies capturing language proficiency (*Mother tongue, Advanced, Intermediate, Beginner*).

The vector *AreaOrigin*_*j*_ includes dummies for the area of origin of the individual specified as follows: *EU-28, EFTA* (EFTA countries), *MENA* (Middle East and North Africa), *Other Europe, NAAO* (North America, Australia and Oceania), *Other Africa, Latin America, ESA* (East and South Asia countries). In line with our expectation that citizenship is of most importance to those migrants who face the highest structural barriers in the labour market, particularly those from less developed parts of the world,^7^ we run separate analyses on the basis of subsamples representing migrants from different origin regions. We distinguish between immigrants from “developed” countries, including those from the *EU-28, EFTA, NAAO*, and immigrants from “developing” countries, including the remaining areas of origin. We recognize that this is a crude distinction and that, had we had better quality information on the precise country of origin of individual respondents (rather than her or his broad region of origin), we would have been able to make a more finely-grained origin country variable measuring development level on a continuous scale (see Peters et al., 2020 for such an approach)^8^.

The vector *MigReason*_*i*_ includes 6 dummy variables specifying the reason for migration immigrants provide in the survey. It contains the following categories: (1) those who declare to have migrated for employment reasons but had not a prearranged job at destination before moving (*Labour)*; (2) those who migrated for study reasons (*Study*); (3) those who migrated to join a family or to form a family (*Family*); (4) those who migrated for the purpose of international protection (*International protection*); and (5) those who migrated for other reasons (*Other reason*). We exclude from the analysis immigrants who declare to have secured employment in the destination country prior to migration. This typology of immigrants are mainly intra-corporate transfers and/or employees recruited through employment agencies and usually do not rely on the classical employment channel and have different career/occupational prospects.

Throughout the baseline estimations we use destination country dummies (ϕ_*c*_) to filter out the effect of all unobserved country-specific factors influencing the labour market outcomes of immigrants. In alternative to this specification, we use several contextual variables to control for the influence of specific destination country characteristics. We include the citizenship policy indicator “The Migrant Integration Policy Index (*MIPEX*) Access to Nationality” measuring the level of legal openness of destination countries regarding access to citizenship. MIPEX is a measure of different policies toward the integration of migrants, where higher scores on a scale from 0 to 100 represent more inclusive migrant integration policies (Niessen et al., 2007). We use an adapted version of the MIPEX subscale for “access to nationality” from the 2013 edition of MIPEX, which only includes those naturalisation criteria which are relevant for first generation migrants. The scores on this subscale are based on the following criteria: eligibility, conditions for acquisition, security of status, and dual nationality.

To check the robustness of the results obtained from MIPEX, we employ an alternative measure based on two indicators developed within the Global Citizenship Observatory: the Citizenship Law (CITLAW) indicators (GLOBALCIT, 2017) and the Citizenship Implementation (CITIMP) indicators (Huddleston, 2013; cf. Huddleston and Vink, 2015 for a comparable approach). Among possible alternative citizenship policy indices, these have the most comparable geographical coverage to MIPEX (Goodman, 2015, p. 1911). From CITLAW, we use ANATORD, which is a general ordinary naturalisation indicator, combining the more specific CITLAW indicators for residence, renunciation requirements, language and civic knowledge requirements, cultural affinity, and economically based naturalisation (Jeffers et al., 2017, p. 7). We calculate the average of the ANATORD and CITIMP measures based on the law in place in 2011, which is the closest available data point comparable to MIPEX 2013 and to the year of data collection for the LFS AHM 2014. The correlation coefficient between MIPEX and ANATORD-CITIMP is 0.62 (see Table A2 in Appendix A) and the Cronbach's alpha statistic is equal to 0.84.

Other destination country variables we use are: *Labour market mobility* measuring the extent legislation and practices support the labour market integration of immigrants; *Unemployment Rate* (data from the World Bank for year 2013) to account for the labour market structure and situation; *Migrants share* (data from Global Bilateral Migration Database for year 2010) which influences the probability of being employed and the typology of jobs available to immigrants. Since the use of mixed-level data may violate the observation's independence (the so-called Moulton problem), we cluster the standard errors at the country level.

In Table 1 we present the descriptive statistics of the employment variable and of the occupational status variable by citizenship, by gender and by the development level of the origin country of immigrants. It is interesting to note that a naturalised immigrant coming from a developing country has the same (unconditional) probability of being employed as a not naturalised immigrant. Conversely, naturalised immigrants coming from developing countries present a higher occupational status (8 points ISEI score) compared to not naturalised immigrants.

**Table 1:** Descriptive statistics of the main dependent variables by citizenship status and development level of the origin country.

	**Employment**			**Occupational status**		
**Immigrants**	**Mean**	**S. D**	**Obs**.	**Mean**	**S. D**	**Obs**.
**Naturalised**	0.635	0.48	3223	37.7	21.7	2045
Developing countries	0.6	0.49	2234	35.4	21	1335
Men	0.68	0.46	849	37.5	20.5	578
Women	0.55	0.5	1385	33.8	21	757
Developed countries	0.72	0.45	989	42	22.5	710
Men	0.91	0.29	364	42	22.1	329
Women	0.61	0.49	625	42.1	22.9	381
**Not naturalised**	0.667	0.47	9611	33.7	21	6400
Developing countries	0.6	0.49	4829	27.4	16	2914
Men	0.7	0.46	2084	28.1	14.8	1452
Women	0.53	0.5	2745	26.6	17.1	1462
Developed countries	0.73	0.44	4782	39	22.7	3486
Men	0.82	0.38	2141	39.2	22.1	1758
Women	0.65	0.47	2641	38.7	23.3	1728

*Source: EU-LFS AHM for year 2014*.

## Results

**Figure 1:**
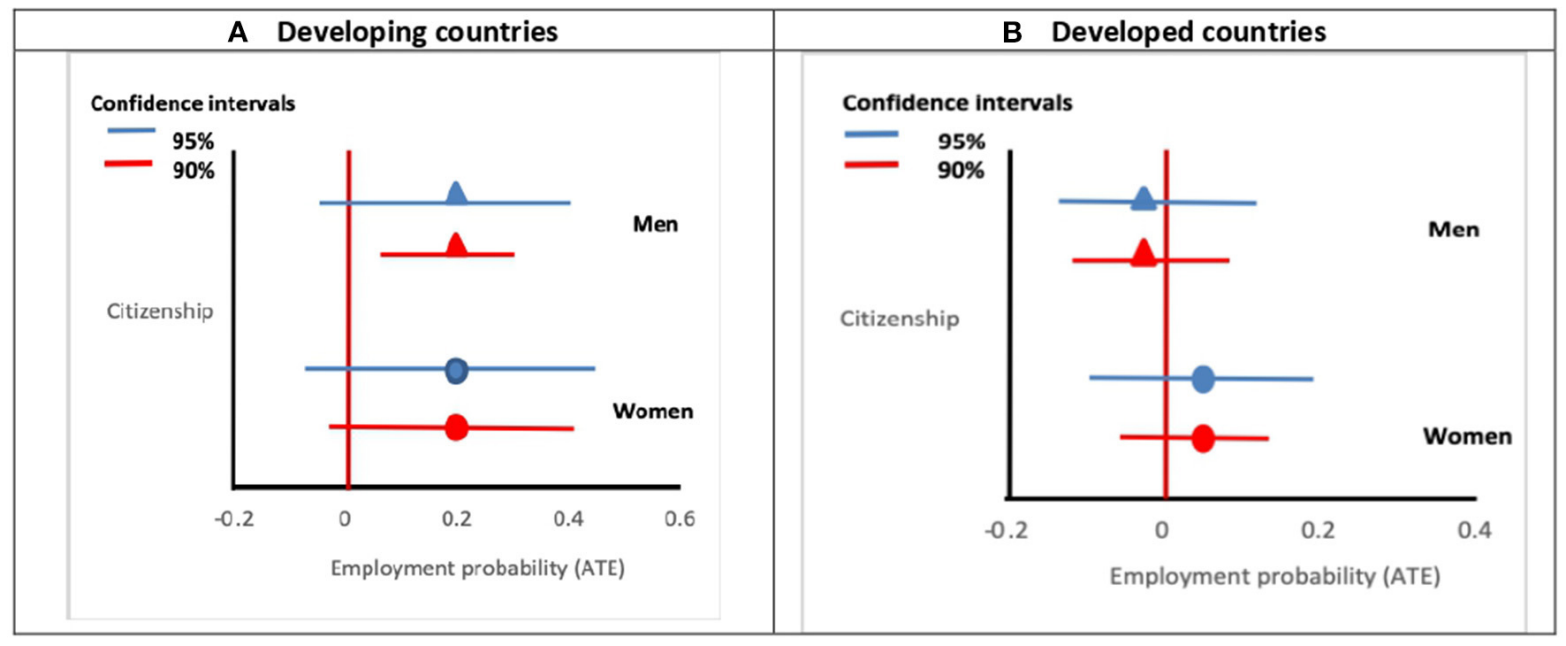
The effect of naturalisation on employment status among immigrants from developing **(A)** and developed countries **(B)**, by gender. Graphs based on the results of Table 1A in the Appendix. The horizontal lines represent the 90 and 95% confidence intervals based on clustered standard errors.

This section summarises the results of the empirical analysis which is conduced separately for men and women and for immigrants coming from developing and developed countries. The choice to estimate separate models by gender is standard in the economic literature as the question of labour market status is generally gender—biased. Instead, the choice to estimate separate models by the development level of the country of origin is less standard in the literature. It is motivated by the different structural obstacles immigrants from developed countries face in the labour market, e.g., less discrimination, few administrative obstacles (free movement for EU and EFTA citizens), compared to immigrants from developing countries. The former type of immigrants is less relevant for the purpose of this analysis also because the reasons to naturalise are often unrelated to the labour market (Vink et al., 2013). Hence, we focus our analysis on immigrants from developing countries. Figure 1 reports the estimated relationship between citizenship and the probability of being employed by gender and by development level of the origin country. Figure 1A shows that naturalisation is positively associated with being employed for men coming from developing countries, but not for women. However, the estimated parameter is moderately significant at 8% level. The probability of being employed for naturalised men is on average 20%^9^ higher than that for non-naturalised.

As expected, we do not find evidence of a significant relationship between citizenship and employment for immigrants coming from developed countries (Figure 1B). One explanation of these results could be the strong signalling effect of citizenship for immigrants from developing countries. By contrast, for immigrants from developed countries, who face a less precarious situation in the labour market, given their presumed higher human capital, as well as a lower chance of statistical discrimination, the effect of signalling is not relevant.

As regards the other covariates (see Table 1A in Appendix A), they mainly show the expected effect on our dependent variables. Generally, human capital variables like education, language proficiency and age (proxy for experience) have a positive effect on the probability of being employed. As expected, individuals migrating to follow their studies show a higher propensity of being employed than those migrating for family reasons show [see models with (a) suffix] while immigrants seeking international protection show a lower propensity as compared to the same category. Generally, more educated individuals and being more proficient in the destination country language is positively associated with being naturalised [see models with (b) suffix]. Economic migrants show a lower probability of naturalisation than individuals migrating for family reasons do, while women seeking international protection are more likely to naturalise. We also find that areas of origin explain a good part of the variation of citizenship acquisition and employment prospects of immigrants. In particular, both men and women immigrants from MENA countries have a lower probability of being employed compared to immigrants from European countries that are not part of EU-28, while immigrants coming from East and South Asia show the opposite result. Results also show that men immigrants from EFTA countries are less likely to naturalised compared to immigrants from EU-28 countries. Conversely, immigrants coming from NAAO countries are more likely to naturalise compared to immigrants coming from EU-28 countries. For these immigrants, naturalisation may serve as a means of overcoming the labour market restrictions and obstacles to free movement in Europe.

Finally, we use the Wald statistic to test for selection bias. The Wald test rejects the null hypothesis of no correlation (ρ) between the error terms in models including only immigrants from developing countries. In models including only immigrants from developed countries the null hypothesis is not rejected at conventional significance levels, meaning that selection is less likely^10^. As argued before, the motivations to naturalise of immigrants from developed countries, and especially of those from EU-27, are often unrelated to the labour market outcomes^11^.

**Figure 2:**
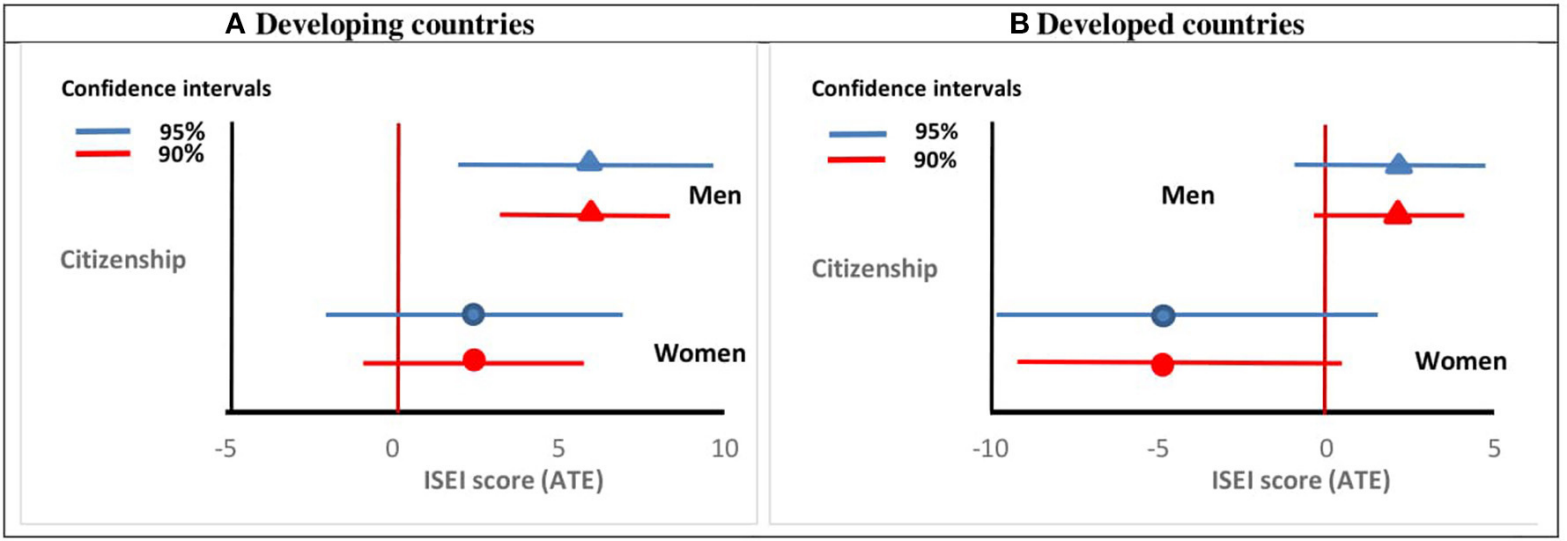
The effect of naturalisation on occupational status among immigrants from developing **(A)** and developed countries **(B)**, by gender. Graphs based on the results of Supplementary Table 1. The horizontal lines represent the 90 and 95% confidence intervals based on clustered standard errors.

Figure 2 explores the relationship between citizenship and occupational status. In these estimations we control for the same individual characteristics as in the case when the dependent variable was employment status. The results show that being a citizen is significantly associated—at 5% level—with a higher job status for migrant men from developing countries but not for women Figure 2A. On average, a naturalised man ranks 5.6 points higher in the ISEI scale than a non-naturalised man does. In substantive terms this, is equivalent to moving from the profession of mason to a professional repairer. This corresponds to a 7% increase on average if we consider the ISEI index range in our sample (11–89). As regards immigrants coming from developed countries, we do not find any association of naturalisation with the occupational status (Figure 2B).

### Specification Check: Institutional Context

Throughout our analysis, we used country dummies to control for all country characteristics that might affect the relationship between citizenship and employment. However, the institutional context, especially the level of accessibility of citizenship, might be one of the factors that influence the relationship between citizenship and employment outcomes among foreign-born residents.

**Figure 3:**
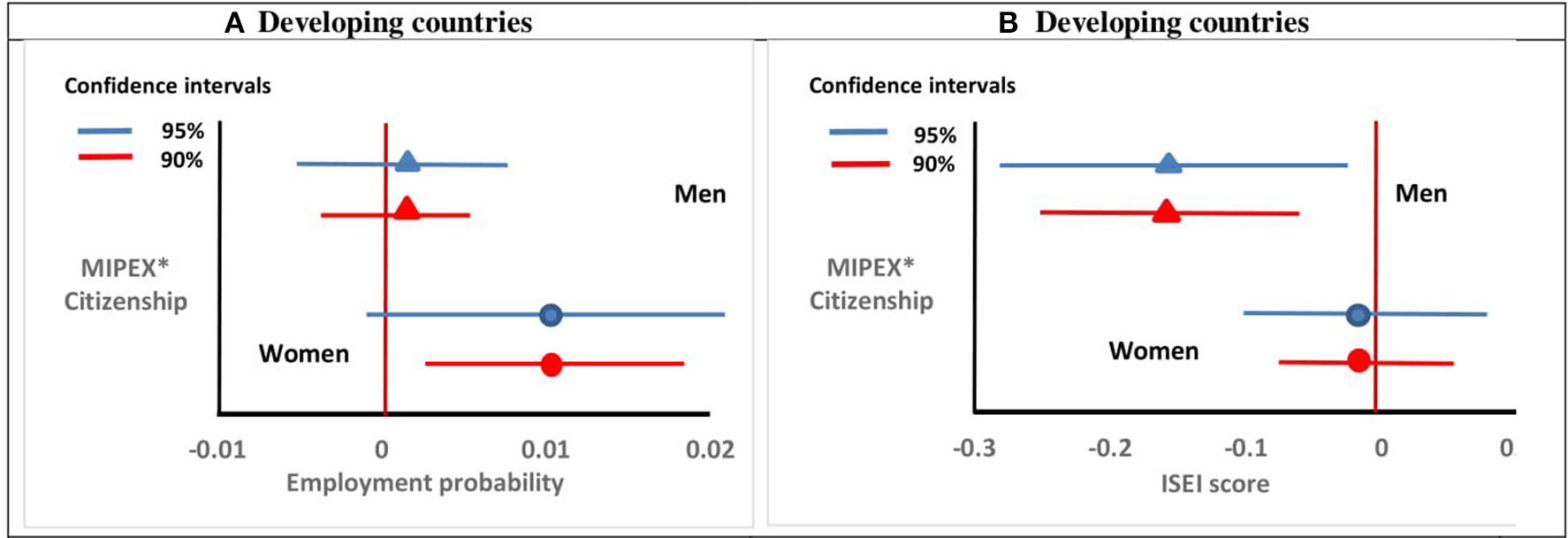
The effect of naturalisation, conditioned by access to citizenship (MIPEX), on employment status **(A)** and occupational status **(B)** among immigrants from developing countries, by gender. Graphs based on the results of Supplementary Table 2. Estimations include variable controlling for labour market integration opportunities each country offers to immigrants *(Labour market mobility)*, the general labour market situation *(Unemployment rate)* and the effect of immigrant's population *(Share Migrants)*. Only immigrants coming from developing countries are considered. The horizontal lines represent the 90 and 95% confidence intervals based on clustered standard errors.

In Figure 3, we present the results of the interaction between the variable *Citizenship* and *MIPEX*. The interaction tests if the relationship between citizenship and employment outcomes is conditioned by access to citizenship. Given the results from our main analyses, we present only the results for immigrants from developing countries. Results show that the effect of citizenship policy is heterogeneous across labour market outcomes and varies by gender. In general, our results suggest that easier access to citizenship increases the positive returns to citizenship in terms of employment. For both men and women, the interaction coefficient is positive but statistically significant at 10 level only for women. This indicates that the positive relationship between citizenship acquisition and employment propensity tends to be stronger under the condition of a less restrictive citizenship policy, but only for women^12^.

One explanation could be the higher investment in specific human capital and language skills in countries where naturalisation is faster, and that immigrants expect to reap these higher returns for a longer period of time. According to Gathmann and Keller (2018), the access to citizenship effect might be less relevant for male immigrants who are more likely to have a permanent work permit and a continuous work history. Indeed, they show that faster access to citizenship more strongly benefited women with no work history who entered the labour market for the first time. Figures 3A,B subsequently presents the results for occupational status. For men, the positive relationship between citizenship and having a better job status is weaker under the condition of having easier access to citizenship. This result is consistent with the “devaluation hypothesis” according to which liberal citizenship policies might “devalue” citizenship as a selection device that signals immigrants' integration in the labour market. For women, the results suggest that access to citizenship does not condition the returns to naturalisation in terms of better jobs. We reproduce these results by using the ANATORD-CITIMP indicator as an alternative measure for the relative accessibility of naturalisation (Supplementary Table 3). Results confirm the positive relationship between more accessible citizenship policy and employment for women. According to this indicator, access to citizenship does not condition the positive effect of citizenship on occupational status for migrant men from developing countries.

## Conclusion

This paper explores the relationship between citizenship and labour market outcomes for foreign-born residents in 13 West European countries. The analysis uses the *ad hoc* module of the European labour force Survey for the year 2014. In order to cope with the selective nature of the naturalisation process, we employ a treatment effect method and a recursive bivariate probit method that account for unobserved characteristics of naturalising immigrants.

Our main finding is that of a positive relationship across these destination countries between citizenship and the probability of employment for immigrant men, as well as between citizenship and occupational status for men. In line with previous findings, we observe that these citizenship premiums only apply to immigrants from developing countries. These findings align with the study of Zwysen (2018), equally based on LFS data but without taking into account the selection of immigrants into citizenship, who finds a slightly positive association of naturalisation with job quality but not with employment.

We find that the effect of citizenship policy is heterogeneous across labour market outcomes and varies by gender. Our analyses show that liberal access to citizenship does not diminish the positive returns on employment from naturalisation. By contrast, in countries where citizenship is relatively easily accessible, the relationship between citizenship and paid employment is stronger for female migrants. However, easier access to citizenship is related with lower returns of naturalisation on occupational status for male immigrants. A tentative explanation for this result may be that a liberal citizenship policy “devalues” the acquisition of citizenship in the eyes of employers and thus serves less as a selection device between immigrants. Further research is needed to better understand why, if at all, such a devaluation hypothesis only seems to hold for occupational status (and not for employment as such) and why only for men (and not for women). Building on our comparative approach as well as the recent work by Helbling et al. (2020), researchers could also further explore the extent to which immigration policies, rather than citizenship policies, condition the citizenship premium in labour markets of destination countries, through the selective impact of admission criteria.

## Data Availability Statement

The dataset analyzed in this study is the Labour Force Survey 2014 *ad hoc* module on the Labour market situation of migrants and their immediate descendants (2014). These data have been obtained from Eurostat. To protect the anonymity of respondents the access to microdata is restricted and data use agreements do not allow us to disclose the individual-level data. Requests to access these data should be directed to Eurostat. Information about the dataset can be obtained here: https://ec.europa.eu/eurostat/documents/1978984/6037334/ESS-agreement-LFS-2014-module-with-annex-EN.pdf.

## Author Contributions

MV, TB, and RH conceived the idea for the paper and developed the theoretical framework. RH prepared the data for the analysis and together with MV implemented the methodology. All authors contributed to the final version of the manuscript.

## Conflict of Interest

The authors declare that the research was conducted in the absence of any commercial or financial relationships that could be construed as a potential conflict of interest.

## Footnotes

^1^Highly skilled workers (managers, technicians, consultants) of multinational enterprises, travelling between affiliates and headquarters are likely to be in this category. Visa costs and reduced flexibility may prevent firms from employing or assigning non-EU immigrants to these positions.

^2^Austria, Belgium, Finland, France, Greece, Italy, Luxembourg, Norway, Portugal, Spain, Sweden, Switzerland, and the United Kingdom.

^3^Reverse causality is another source of endogeneity. We deal with this issue in Appendix B.

^4^The treatment effect model is used because occupational status is a continuous variable. Both methods assume that standard errors of the simultaneously estimated equations are jointly normally distributed. The score test (Murphy, 2007; Chiburis et al., 2012) rejects misspecification of the recursive bivariate probit model at 5%.

^5^Employed individuals are categorised following the definition of ILO.

^6^The ISEI score ranks worker occupations into a scale which varies from 11 (subsistence farming) to 89 (medical occupations). It is computed as weighted averages of standardised measures of income and education of incumbents in each ISCO 08 classification of occupations. Hence, ISEI scale is suitable for across countries comparisons.

^7^Research also suggests that naturalisation propensity differs strongly between migrants from developed and developing countries (Vink et al., 2013).

^8^Note that Japan and South Korea are considered in the “developing” countries category due to EUROSTAT categorisation of these countries in the East Asia category together with other developing countries.

^9^It is measured as the average treatment effect (ATE) and corresponds to the marginal effect of citizenship (dichotomous variable) on the probability of being employed across the 13 countries considered in the analysis. The magnitude of this effect may depend on the number of observations for each country in our sample. As the magnitude of the cross-country effect is not a primary interest of this study and given that population weighting could artificially increase the standard errors, we do not use population weights in our estimations.

^10^We estimate these models using the probit model. Results do not change significantly from those reported in Table 1A. Results are available upon request.

^11^Selection is absent when unobserved characteristics influencing the propensity to naturalise are not correlated to employment.

^12^The parameter is weakly significant at 7.5% level.
