# Supplementary material for: Immigrant Naturalisation, Employment and Occupational Status in Western Europe
Source: Front Sociol. 2020 Dec 16;5:70. doi: 10.3389/fsoc.2020.00070 (PMC8022582; doi:10.3389/fsoc.2020.00070)
Supplement: Supplementary file 1 [file Data_Sheet_1.docx]

Supplementary material of the paper: **Immigrant naturalisation, employment and occupational status in Western Europe**

| **Table 1SM.** The effect of naturalisation on occupational status | | | | |  |  |  |  |
| --- | --- | --- | --- | --- | --- | --- | --- | --- |
|  | Model (1a) | (1b) | Model (2a) | (2b) | Model (3a) | (3b) | Model (4a) | (4b) |
| VARIABLES | Occ. status | Selection | Occ. status | Selection | Occ. status | Selection | Occ. status | Selection |
|  | Men | | Women | | Men | | Women | |
|  | Developing | | Developing | | Developed | | Developed | |
| Citizenship | 5.558*** |  | 2.241 |  | 2.675 |  | -5.095 |  |
|  | (1.537) |  | (1.858) |  | (1.887) |  | (3.887) |  |
| Age | -0.0822 | 0.0427 | -0.717** | 0.0415 | 0.889** | 0.105 | 0.451 | 0.0587 |
|  | (0.232) | (0.0300) | (0.349) | (0.0328) | (0.353) | (0.0962) | (0.392) | (0.0568) |
| Age squared | 0.000838 | -0.000382 | 0.00624 | -0.000446 | -0.00937** | -0.00123 | -0.00489 | -0.000680 |
|  | (0.00264) | (0.000404) | (0.00403) | (0.000381) | (0.00382) | (0.00106) | (0.00471) | (0.000603) |
| Married | -1.034 | 0.400*** | 1.353 | 0.342*** | -1.600* | 0.420*** | 0.0130 | 0.463*** |
|  | (1.078) | (0.125) | (0.948) | (0.0977) | (0.958) | (0.0944) | (0.942) | (0.137) |
| Divorced | -2.075 | 0.291* | -0.694 | 0.145** | -5.332*** | 0.276** | -1.100 | 0.441*** |
|  | (1.487) | (0.166) | (1.588) | (0.0649) | (1.540) | (0.140) | (1.165) | (0.110) |
| Years of Residence | -0.652 | 0.267*** | 0.690 | 0.228** | -0.387 | 0.165*** | 2.291*** | 0.230*** |
|  | (0.873) | (0.0874) | (0.616) | (0.0939) | (0.545) | (0.0503) | (0.660) | (0.0768) |
| Years of Residence 2 | 0.0309 | -0.00672* | -0.0246 | -0.00441 | 0.0185 | -0.00308 | -0.0795*** | -0.00434 |
|  | (0.0320) | (0.00344) | (0.0240) | (0.00365) | (0.0208) | (0.00190) | (0.0221) | (0.00351) |
| Medium edu. class | 2.874*** | 0.0857 | 1.891*** | 0.183*** | 1.649 | 0.0616 | 4.695** | 0.189 |
|  | (0.924) | (0.0701) | (0.723) | (0.0675) | (1.150) | (0.167) | (2.010) | (0.150) |
| High edu. class | 16.09*** | 0.155 | 16.93*** | 0.328*** | 20.79*** | -0.0541 | 24.76*** | 0.146 |
|  | (2.572) | (0.109) | (3.086) | (0.0961) | (3.453) | (0.171) | (3.596) | (0.172) |
| Labour | 5.365** | -0.247 | 7.474** | -0.00722 | 8.155*** | -0.545*** | 5.689*** | -0.178 |
|  | (2.197) | (0.211) | (3.064) | (0.0959) | (1.410) | (0.124) | (1.363) | (0.133) |
| Study | -4.737*** | 0.0533 | -2.094 | 0.296*** | -5.144* | -0.0666 | 2.446 | 0.266 |
|  | (0.846) | (0.240) | (1.573) | (0.0858) | (2.692) | (0.272) | (1.660) | (0.343) |
| International | -0.282 | -0.233 | 0.917 | -0.0531 | 0.0935 | -0.273** | 1.904 | -0.206 |
| protection | (1.116) | (0.172) | (1.334) | (0.0998) | (1.595) | (0.117) | (1.915) | (0.173) |
| Other reason | -0.889 | 0.130 | 0.596 | 0.196* | 0.747 | 0.313* | -1.959 | 0.419*** |
|  | (1.540) | (0.159) | (0.978) | (0.106) | (0.807) | (0.185) | (2.165) | (0.144) |
| Intermediate | 1.926 | 0.407** | 6.057*** | 0.328*** | 6.326*** | 0.510*** | 3.126 | 0.701*** |
|  | (1.345) | (0.195) | (1.576) | (0.106) | (1.398) | (0.143) | (3.252) | (0.115) |
| Advanced | 3.565** | 0.707*** | 8.710*** | 0.547** | 9.338*** | 0.858*** | 9.143*** | 0.887*** |
|  | (1.603) | (0.172) | (1.963) | (0.249) | (1.604) | (0.256) | (2.370) | (0.152) |
| Mother tongue | -0.0822 | 0.0427 | -0.717** | 0.0415 | 0.889** | 0.105 | 0.451 | 0.0587 |
|  | (0.232) | (0.0300) | (0.349) | (0.0328) | (0.353) | (0.0962) | (0.392) | (0.0568) |
| Other Africa | 1.261 | -0.043 | -1.59 | 0.113 |  |  |  |  |
|  | (0.884) | (0.097) | (1.214) | (0.164) |  |  |  |  |
| MENA | 2.794 | 0.26 | 0.272 | 0.259 |  |  |  |  |
|  | (1.281) | (0.159) | (2.032) | (0.229) |  |  |  |  |
| ESA | 7.63*** | -0.002 | 8.1*** | -0.459*** |  |  |  |  |
|  | (1.996) | (0.235) | (2.71) | (0.178) |  |  |  |  |
| Latin America | 2.42 | 0.451* | -0.151 | 0.193 |  |  |  |  |
|  | (1.775) | (0.250) | (0.744) | (0.241) |  |  |  |  |
| EFTA |  |  |  |  | 3.871 | -0.719*** | 14.21*** | -1.052 |
|  |  |  |  |  | (4.44) | (0.137) | (1.95) | (0.845 |
| NAAO |  |  |  |  | -1.425 | 1.15*** | -1.974 | 1.18*** |
|  |  |  |  |  | (0.994) | (0.302) | (2.957) | (0.366) |
| Constant | 29.94*** | -4.867*** | 33.24*** | -4.930*** | 11.71 | -6.162*** | -0.718 | -6.339*** |
|  | (6.230) | (0.799) | (10.02) | (0.691) | (9.043) | (2.075) | (11.06) | (1.363) |
| Wald test (Rho=0) | Rejected | | Not rejected | | Not rejected | | Rejected | |
| Observations | 2,030 | 2,030 | 2,219 | 2,219 | 2,087 | 2,087 | 2,109 | 2,109 |
| Dependent variables: Occupational status. Reference categories are: for reason to migrate is family reason; for marital status is single; for education is low education class; for language is beginner; for country areas are Other Europe for developing countries and EU-28 for developed countries.. Destination country dummies are included. Errors (in parentheses) are clustered at country level. *** p<0.01, ** p<0.05, * p<0.1 | | | | | | | | |

| **Table 2SM.** The effect of naturalisation, conditioned by access to citizenship (MIPEX), on employment status and occupational status among immigrants coming from developing countries, by gender. | | | | | | | | |
| --- | --- | --- | --- | --- | --- | --- | --- | --- |
|  | Model (1a) | (1b) | Model (2a) | (2b) | Model (3a) | (3b) | Model (4a) | (4b) |
| VARIABLES | Employment | Citizenship | Employment | Citizenship | Occ. status | Citizenship | Occ. status | Citizenship |
|  | Men | | Women | | Men | | Women | |
| Citizenship | 0.920** |  | 0.116 |  | 15.70*** |  | 5.781 |  |
|  | (0.409) |  | (0.648) |  | (5.107) |  | (4.832) |  |
| MIPEX | -0.0133* | 0.0302*** | -0.00126 | 0.0224** | 0.102*** | 0.0303*** | 0.0139 | 0.0229** |
|  | (0.00784) | (0.0104) | (0.00655) | (0.0103) | (0.0227) | (0.0109) | (0.0435) | (0.0110) |
| Citizenship*MIPEX | 0.000748 |  | 0.00968* |  | -0.168** |  | -0.0213 |  |
|  | (0.00378) |  | (0.00543) |  | (0.0712) |  | (0.0661) |  |
| Labour market mobility | -0.00143 | -0.00690 | 0.00695*** | -0.00891 | -0.0673 | -0.00841 | -0.0827 | -0.00711 |
|  | (0.00244) | (0.00947) | (0.00205) | (0.0116) | (0.0459) | (0.0101) | (0.0566) | (0.0139) |
| Unemployment | -0.0245** | -0.0106 | -0.0116** | -0.00165 | -0.265*** | -0.0164 | -0.536*** | 0.0128 |
|  | (0.00995) | (0.0167) | (0.00452) | (0.0157) | (0.0794) | (0.0199) | (0.104) | (0.0223) |
| Migrants share | -0.00851 | 0.00105 | 0.0222*** | -0.0141 | 0.224** | 0.00765 | 0.347*** | -0.0123 |
|  | (0.0134) | (0.0376) | (0.00462) | (0.0261) | (0.0954) | (0.0373) | (0.133) | (0.0281) |
| Constant | 0.0725 | -4.340*** | -4.022*** | -3.699*** | 28.58*** | -4.364*** | 40.12*** | -4.179*** |
|  | (1.145) | (1.062) | (1.046) | (1.050) | (7.003) | (1.275) | (9.948) | (1.348) |
| Wald test (Rho=0) | Rejected | | Not rejected | | Rejected | | Not rejected | |
| Observations | 2,933 | 2,933 | 4,130 | 4,130 | 2,030 | 2,030 | 2,219 | 2,219 |
| Dependent variable: Employment and occupational status. All baseline variables are included in the estimations. Only immigrants coming from developing countries are considered. Errors (in parentheses) are clustered at country level. *** p<0.01, ** p<0.05, * p<0.1 | | | | | | | | |

| **Table 3SM.** The effect of naturalisation, conditioned by access to citizenship (ANATORD-CITIMP), on employment status and occupational status among immigrants coming from developing countries, by gender. | | | | | | | | |
| --- | --- | --- | --- | --- | --- | --- | --- | --- |
|  | Model (1a) | (1b) | Model (2a) | (2b) | Model (3a) | (3b) | Model (4a) | (4b) |
| VARIABLES | Employment | Citizenship | Employment | Citizenship | Occ. status | Citizenship | Occ. status | Citizenship |
|  | Men | | Women | | Men | | Women | |
| Citizenship | 0.808* |  | -0.315 |  | 10.05** |  | 7.366* |  |
|  | (0.425) |  | (0.867) |  | (3.914) |  | (4.251) |  |
| ANATORD-CITIMP | -1.817** | 4.242*** | -0.404 | 3.619*** | 11.04** | 4.445*** | 14.70** | 4.156*** |
|  | (0.787) | (0.732) | (0.930) | (0.726) | (4.668) | (0.742) | (5.812) | (0.844) |
| Citizenship*ANATORD-CITIMP | 0.349 |  | 1.644*** |  | -7.993 |  | -7.573 |  |
|  | (0.430) |  | (0.492) |  | (6.872) |  | (5.302) |  |
| Labour market mobility | -0.00235 | -0.00274 | 0.00804*** | -0.00536 | -0.0568 | -0.00286 | -0.0860 | -0.00459 |
|  | (0.00199) | (0.00587) | (0.00207) | (0.00816) | (0.0442) | (0.00609) | (0.0560) | (0.00945) |
| Unemployment | -0.0102 | -0.0406*** | -0.0122 | -0.0265*** | -0.339*** | -0.0454*** | -0.602*** | -0.0142 |
|  | (0.0117) | (0.00714) | (0.00907) | (0.00911) | (0.0829) | (0.00972) | (0.0839) | (0.0147) |
| Migrants share | -0.00448 | 0.00129 | 0.0230*** | -0.0155 | 0.222*** | 0.0136 | 0.355** | -0.00954 |
|  | (0.00939) | (0.0325) | (0.00552) | (0.0222) | (0.0725) | (0.0332) | (0.142) | (0.0252) |
| Constant | 0.292 | -5.155*** | -3.993*** | -4.611*** | 27.91*** | -5.721*** | 31.65*** | -5.124*** |
|  | (1.155) | (0.888) | (1.336) | (0.750) | (6.898) | (0.896) | (9.419) | (1.261) |
| Wald test (Rho=0) | Rejected | | Not rejected | | Rejected | | Not rejected | |
| Observations | 2,933 | 2,933 | 4,130 | 4,130 | 2,030 | 2,030 | 2,219 | 2,219 |
| Dependent variable: Employment and occupational status. All baseline variables are included in the estimations. Only immigrants coming from developing countries are considered. Errors (in parentheses) are clustered at country level. *** p<0.01, ** p<0.05, * p<0.1 | | | | | | | | |

| **Table 4SM** Descriptive statistics | | | | | | |  |
| --- | --- | --- | --- | --- | --- | --- | --- |
|  | **Total** | | **Female** | | **Male** | | |
| **VARIABLES** | **Mean** | **S.D** | **Mean** | **S.D** | **Mean** | **S.D** | |
| Citizenship | 0.25 | 0.43 | 0.27 | 0.44 | 0.22 | 0.42 | |
| Occupational status (ranges 11-89) | 34.7 | 21 | 34.1 | 21 | 35.3 | 20.3 | |
| Employed | 0.66 | 0.47 | 0.58 | 0.49 | 0.76 | 0.43 | |
| Developing Country | 0.55 | 0.49 | 0.56 | 0.5 | 0.54 | 0.49 | |
| Female | 0.42 | 0.49 |  |  |  |  | |
| Age (years) | 42.3 | 8.3 | 42.3 | 8.5 | 42.3 | 8.1 | |
| Years of residence (years) | 12.4 | 3.5 | 12.4 | 3.5 | 12.4 | 3.5 | |
| Married | 0.7 | 0.45 | 0.68 | 0.47 | 0.74 | 0.43 | |
| Single | 0.17 | 0.37 | 0.15 | 0.36 | 0.18 | 0.39 | |
| Divorced/separated | 0.13 | 0.33 | 0.17 | 0.37 | 0.07 | 0.26 | |
| **Education level** |  |  |  |  |  |  | |
| Low education | 0.35 | 0.47 | 0.34 | 0.47 | 0.37 | 0.48 | |
| Medium education | 0.35 | 0.48 | 0.36 | 0.48 | 0.35 | 0.48 | |
| High education | 0.30 | 0.29 | 0.3 | 0.46 | 0.28 | 0.45 | |
| **Migration reason** |  |  |  |  |  |  | |
| Labour | 0.41 | 0.49 | 0.31 | 0.46 | 0.52 | 0.49 | |
| Family | 0.39 | 0.48 | 0.50 | 0.50 | 0.23 | 0.41 | |
| Study | 0.06 | 0.24 | 0.05 | 0.23 | 0.08 | 0.25 | |
| International protection | 0.06 | 0.24 | 0.05 | 0.22 | 0.08 | 0.27 | |
| Other reason | 0.08 | 0.27 | 0.07 | 0.49 | 0.09 | 0.28 | |
| **Language proficiency** |  |  |  |  |  |  | |
| Beginner | 0.11 | 0.31 | 0.12 | 0.32 | 0.10 | 0.30 | |
| Intermediate | 0.29 | 0.45 | 0.28 | 0.45 | 0.31 | 0.46 | |
| Advanced | 0.37 | 0.48 | 0.36 | 0.48 | 0.37 | 0.48 | |
| Mother tongue | 0.23 | 0.41 | 0.24 | 0.42 | 0.19 | 0.40 | |
| **Total number** | **12834** | | **7396** | | **5438** | | |
| Population weights are used. Source: EU-LFS Ad Hoc Module for year 2014. | | | | | | | |
|  | | | | | | | |

| **Table 5SM** Distribution of immigrants by country of destination and gender | | | |
| --- | --- | --- | --- |
|  |  | |  |
| **Country** | **Female** | **Male** | **Total** |
| Austria | 328 | 223 | 551 |
| Belgium | 487 | 376 | 863 |
| Switzerland | 250 | 151 | 401 |
| Spain | 887 | 657 | 1544 |
| Finland | 129 | 99 | 228 |
| France | 359 | 272 | 631 |
| Greece | 505 | 450 | 955 |
| Italy | 1882 | 1290 | 3172 |
| Luxembourg | 427 | 279 | 706 |
| Norway | 204 | 185 | 389 |
| Portugal | 212 | 144 | 356 |
| Sweden | 356 | 280 | 634 |
| United Kingdom | 1370 | 1032 | 2402 |
| **Total** | **7396** | **5438** | **12834** |
| Source: EU-LFS AHM for year 2014. | | | |
