# Supplementary material for: Immigrant Naturalisation, Employment and Occupational Status in Western Europe
Source: Front Sociol. 2020 Dec 16;5:70. doi: 10.3389/fsoc.2020.00070 (PMC8022582; doi:10.3389/fsoc.2020.00070)
Supplement: Supplementary file 2 [file Data_Sheet_1.pdf]

APPENDIX A

Table 1A. The effect of naturalisation on employment status among immigrants from developing and developed countries, by gender.

VARIABLES	Model (1a) Employment	(1b) Citizenship	Model (2a) Employment	(2b) Citizenship	Model (3a) Employment	(3b) Citizenship	Model (4a) Employment	(4b) Citizenship
	Men Developing		Women Developing		Men Developed		Women Developed	
Citizenship (ATE)	0.203*		0.2		-0.021		0.07	
Citizenship	0.738*		0.634		-0.0769		0.210	
	(0.422)		(0.532)		(0.547)		(0.540)	
Age	0.0509	0.0355	0.122***	0.0430***	0.0958***	0.0691	0.157***	0.0112
	(0.0364)	(0.0403)	(0.0272)	(0.0153)	(0.0241)	(0.0750)	(0.0211)	(0.0302)
Age squared	-0.000883**	-0.000335	-0.00139***	-0.00045***	-0.00140***	-0.000839	-0.00180***	-8.98e-05
	(0.000401)	(0.000481)	(0.000304)	(0.000176)	(0.000252)	(0.000795)	(0.000240)	(0.000355)
Married	0.192*	0.345***	-0.313***	0.378***	0.201***	0.436***	-0.279**	0.531***
	(0.106)	(0.130)	(0.0939)	(0.0878)	(0.0541)	(0.100)	(0.111)	(0.113)
Divorced	0.357**	0.169	-0.0904	0.208***	0.0211	0.297*	-0.140	0.498***
	(0.146)	(0.134)	(0.0917)	(0.0744)	(0.0727)	(0.157)	(0.106)	(0.116)
Years of Residence	0.0600	0.257***	0.130	0.239***	0.00986	0.172***	0.0661	0.186***
	(0.0596)	(0.0815)	(0.0933)	(0.0686)	(0.0534)	(0.0410)	(0.0414)	(0.0591)
Years of Residence 2	-0.00254	-0.00625*	-0.00581*	-0.00555**	0.000273	-0.00335*	-0.00284	-0.00330
	(0.00182)	(0.00323)	(0.00313)	(0.00267)	(0.00191)	(0.00173)	(0.00210)	(0.00263)
Medium edu. class	0.0873	0.113**	0.0862	0.138***	0.132**	0.0553	0.148*	0.191
	(0.0784)	(0.0517)	(0.0674)	(0.0516)	(0.0568)	(0.157)	(0.0815)	(0.120)
High edu. class	0.106	0.236***	0.191**	0.312***	0.397***	0.0109	0.450***	0.109
	(0.0767)	(0.0682)	(0.0874)	(0.0821)	(0.115)	(0.146)	(0.102)	(0.0987)
Labour	0.132	-0.263***	0.602***	-0.231**	0.189	-0.349***	0.554***	-0.410***
	(0.0834)	(0.0921)	(0.0416)	(0.117)	(0.117)	(0.0451)	(0.0408)	(0.0814)
Study	0.376***	-0.248	0.226***	-0.108	0.239*	-0.559***	0.226**	-0.205*
	(0.137)	(0.165)	(0.0790)	(0.0848)	(0.128)	(0.110)	(0.0925)	(0.117)
International protection	-0.251**	0.0887	-0.101	0.194*	-0.646***	0.0822	-0.271	0.231
	(0.103)	(0.137)	(0.0759)	(0.117)	(0.175)	(0.283)	(0.216)	(0.248)
Other reason	0.148	-0.187**	0.150**	-0.00763	0.121	-0.281**	0.171	-0.218
	(0.125)	(0.0947)	(0.0615)	(0.107)	(0.175)	(0.111)	(0.131)	(0.137)
Intermediate	0.278***	0.102	0.363***	0.178**	0.246**	0.233	0.346***	0.362***
	(0.0954)	(0.129)	(0.0822)	(0.0850)	(0.0962)	(0.151)	(0.0943)	(0.127)
Advanced	0.318***	0.342***	0.572***	0.334***	0.351***	0.451***	0.464***	0.712***
	(0.111)	(0.127)	(0.117)	(0.0833)	(0.0869)	(0.138)	(0.113)	(0.183)
Mother tongue	0.269**	0.631***	0.387*	0.647***	0.335***	0.765***	0.524***	0.805***
	(0.130)	(0.172)	(0.216)	(0.204)	(0.0980)	(0.239)	(0.166)	(0.197)
Other Africa	0.011	0.057	0.0253	0.219*				
	(0.107)	(0.112)	(0.072)	(0.118)				
MENA	-0.299***	0.264	-0.521***	0.271				
	(0.082)	(0.203)	(0.055)	(0.199)				
ESA	0.523***	0.002	0.426***	-0.202				
	(0.172)	(0.265)	(0.121)	(0.195)				
Latin America	0.001	0.56**	-.0101	0.365				
	(0.127)	(0.287)	(0.071)	(0.223)				
EFTA					-0.287	-0.704***	-0.289	-0.84
					(0.273)	(0.135)	(0.292)	(0.603)
NAAO					0.164	1.094***	-0.328	1.085***
					(0.1928)	(0.2906)	(0.221)	(0.359)
Constant	-0.627	-4.619***	-3.381***		-1.207*	-5.424***	-3.483***	-4.877***
	(1.049)	(1.014)	(1.119)		(0.637)	(1.634)	(0.467)	(0.639)
Wald test (Rho=0)	Rejected		Rejected		Not rejected		Not rejected	
Observations	2,933	2,933	4,130	4,130	2,505	2,505	3,266	3,266

Dependent variables: Employment status. Reference categories are: for reason to migrate is family reason; for marital status is single; for education is low education class; for language is beginner; for country areas are Other Europe for developing countries and EU-28 for developed countries. Destination country dummies are included. Origin area dummies are included. Robust standard errors in parentheses. *** p<0.01, ** p<0.05, * p<0.1

Table 2A. Correlation matrix: macro-level variables

	(1)	(2)	(3)	(4)	(5)
(1) Labour market mobility	1				
(2) MIPEX Access to Nationality (1st gen)	-0.053	1			
(3) ANATORD-CITIMP	-0.156	0.62	1		
(4) Unemployment share	0.07	-0.426	0.017	1	
(5) Share migrants	-0.35	-0.198	0.006	-0.07	1

Source: EU-LFS Ad-Hoc Module for year 2014

APPENDIX B

Endogeneity concerns

In order to naturalise, in some countries, immigrants have to demonstrate that they are economically self-sufficient. Indeed, being employed and/or earning a given threshold level of income is a precondition for naturalisation. For example, in Italy, a candidate should demonstrate to have earned in each of 3 years preceding the naturalisation application at least an income of around 8300 Euros. Clearly, explicit requirements on economic self-sufficiency give rise to endogeneity bias if the aim is to estimate the effect of citizenship on labour market outcomes. This may imply reverse causality in our analyses if income - and therefore employment - is both a precondition for naturalisation and an expected result of citizenship status. In order to confirm that our baseline results are not affected by endogeneity, we exclude from the analysis those countries that have explicit economic requirements in order to apply for citizenship.

Figure 1B replicates the baseline estimations presented in Figure 1 and Figure 2 with the difference that countries that have explicit economic requirements such as Austria, Italy and Finland are now excluded. The baseline results are confirmed for men with a slight increase in the magnitude of the relationship.

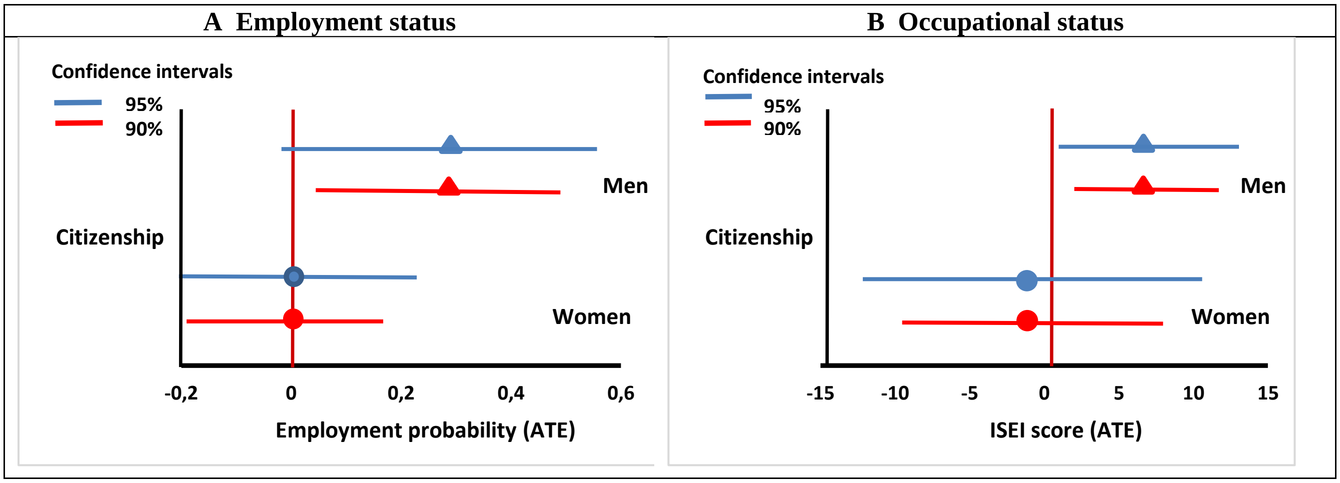

Figure 1B The effect of naturalisation on employment status (A) and occupational status (B), by gender. Only immigrants coming from developing countries are considered. The horizontal lines represent the 90% and 95% confidence intervals based on clustered standard errors.

Figure 2B replicates estimations of Figure 3. Overall, results are similar to those obtained in Figure 3, except the relationship between access to citizenship and employment of women that remains positive but turns not significant at standard levels. The loss in significance could be due to the decrease in variability following the exclusion of 3 important countries from the estimations. These countries represent around one third of the overall number of observations (1400 of 4130 observations).

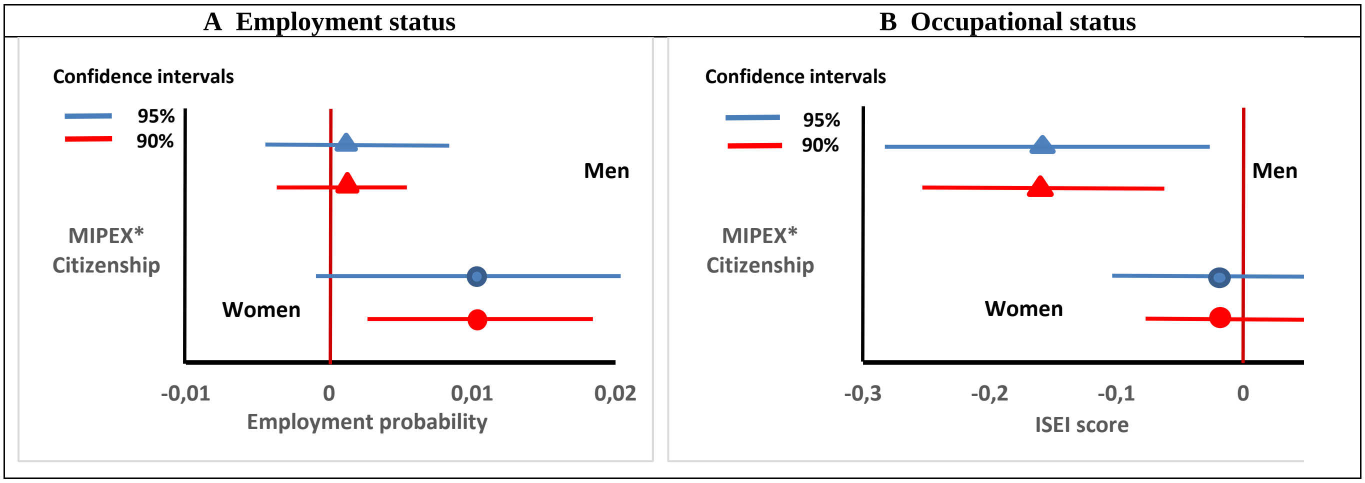

Figure 2B. The effect of naturalisation, conditioned by access to citizenship (MIPEX), on employment status (A) and occupational status (B) among immigrants from developing countries, by gender. The horizontal lines represent the 90% and 95% confidence intervals based on clustered standard errors.
